# High-Performance Perovskite Solar Cells and Modules Fabricated by Slot-Die Coating with Nontoxic Solvents

**DOI:** 10.3390/nano13111760

**Published:** 2023-05-29

**Authors:** Chia-Feng Li, Hung-Che Huang, Shih-Han Huang, Yu-Hung Hsiao, Priyanka Chaudhary, Chun-Yu Chang, Feng-Yu Tsai, Wei-Fang Su, Yu-Ching Huang

**Affiliations:** 1 Department of Materials Science and Engineering, National Taiwan University, Taipei 10617, Taiwan; d10527005@ntu.edu.tw (C.-F.L.); r06527061@ntu.edu.tw (H.-C.H.); ftsai@ntu.edu.tw (F.-Y.T.); suwf@ntu.edu.tw (W.-F.S.); 2 Department of Materials Engineering, Ming Chi University of Technology, New Taipei City 24301, Taiwan; d05527006@ntu.edu.tw (S.-H.H.); u08187148@mail2.mcut.edu.tw (Y.-H.H.); priyanka@mail.mcut.edu.tw (P.C.); 3 Sumei Chemical Co., Ltd., Taoyuan City 32849, Taiwan; october32019@gmail.com; 4 Organic Electronics Research Center, Ming Chi University of Technology, New Taipei City 24301, Taiwan; 5 Center for Plasma and Thin Film Technologies, Ming Chi University of Technology, New Taipei City 24301, Taiwan

**Keywords:** perovskite solar cells, slot-die coating, interface, module, nontoxic solvent

## Abstract

Energy shortage has become a global issue in the twenty-firt century, as energy consumption grows at an alarming rate as the fossil fuel supply exhausts. Perovskite solar cells (PSCs) are a promising photovoltaic technology that has grown quickly in recent years. Its power conversion efficiency (PCE) is comparable to that of traditional silicon-based solar cells, and scale-up costs can be substantially reduced due to its utilization of solution-processable fabrication. Nevertheless, most PSCs research uses hazardous solvents, such as dimethylformamide (DMF) and chlorobenzene (CB), which are not suitable for large-scale ambient operations and industrial production. In this study, we have successfully deposited all of the layers of PSCs, except the top metal electrode, under ambient conditions using a slot-die coating process and nontoxic solvents. The fully slot-die coated PSCs exhibited PCEs of 13.86% and 13.54% in a single device (0.09 cm^2^) and mini-module (0.75 cm^2^), respectively.

## 1. Introduction

Perovskite materials have been used in the past due to their superior photoelectric properties, including low defect density, long carrier diffusion length, and high absorption coefficient in the visible light spectrum. The field of perovskite solar cells (PSCs) has progressed rapidly in the last decade [1,2,3,4]. At laboratory scale, PSCs have currently achieved the power conversion efficiency (PCE) of 25.70% [5], which is comparable to that of the conventional silicon-based solar cells. Therefore, PSCs are considered to have great potential for commercial mass production [6,7]. However, most of the PSCs with high PCEs are still at the laboratory level, where fabrication is achieved by the spin coating process. The spin coating process is not suitable for large-area PSC manufacture due to the low material utilization and limitations for large-area coating. To realize the commercial applications of PSCs, many research groups have developed various techniques for large-area PSC manufacture, such as inkjet printing [8], blade coating [9], slot-die coating [10,11,12], and spray coating [13,14].

Among the process methods, slot-die coating has been extensively developed in scalable PSCs. Mathews et al. achieved a module PCE of 16.22% by adding an alkali additive [15]. Lee et al. introduced an efficient additive with a carboxyl group for slot-die coated PSC production, reaching a PCE of 14.63% [16]. Luoyi et al. reported a PCE of 14.55% for fully slot-die coated n-i-p PSCs [17], and Thai et al. reported a PCE of 11.70% for a fully slot-die coated p-i-n PSC module [18]. Although good progress has been made in large-area PSCs, the use of toxic solvents in these studies has hindered the commercialization of PSCs. In our previous work [11], we added butanol in a nontoxic solvent system, the mixing solution of dimethyl sulfoxide (DMSO) and gamma-butyrolactone (GBL), to control the dry rate and improve the crystallization of the perovskite films. However, the long-term use of GBL can still be harmful to humans. Therefore, the development of new nontoxic solvents system for large-area slot-die coated PSCs is necessary.

Recently, several studies have been carried out to enhance the performance of PSCs. Li et al. introduced a binary-mixed organic ETL, namely PCBM and m-ITIC, to improve the PCE and stability of the PSCs [19]. Layered Bi-containing compounds, such as BiO_2_S and BiO_2_Se, have great potential in photoelectric applications [20]. Yu. used 2D Bi_2_O_2_Se nanoflakes as an ETL in PSCs due to their ultrahigh mobility and high carrier concentration [21]. Deng et al. combined 2D halide perovskite nanosheets with 3D MAPbI_3_, which greatly improved the stability and increased the PCE to 20% [22]. In addition to the PCE, the stability of PSCs is also a critical issue. Top metal electrodes play a key role in large-area perovskite solar cells, as they are the electrical contacts for the device and facilitate the transport of charge carriers. The previous literature has investigated the interaction of electrode-induced degradation pathways of perovskite layers [23]. Their results show that the PCE of Ag-based PSCs decreases rapidly compared to Au-based PSCs because of the formation of AgI, which leads to corrosion of the perovskite layer. Furthermore, the effect of ion migration-induced electrode degradation on the stability of PSCs was also investigated systematically [24]. For the power generation systems, PSCs need to be combined with energy storage devices, such as batteries. Rechargeable batteries based on multivalent ions have been developed for high energy density storage systems. Lee et al. reported an aluminum–graphite dual-ion battery with high reversibility and high energy density [25]. Cheng et al. proposed a rechargeable Ca-ion battery with a highly reversible electrochemical reaction at room temperature [26]; Huang et al. designed 1D V_3_S_4_@NC fibers for a sodium-ion battery [27]. All the batteries have great potential for future integration with PSCs as complete power generation systems.

In this work, we developed fully slot-die coated PSCs mainly with nontoxic solvents under ambient conditions, in which the hole transport layer (HTL), the perovskite active layer, the electron transport layer (ETL), and work function (WF) modified layer were optimized. With a device area of 0.09 cm^2^, the PCE of the PSC fabricated from slot-die coating reached 13.86%, which is similar to that of the PSCs fabricated from the spin coating process. We further expanded the device area and obtained a mini-module of 0.75 cm^2^ with a PCE of 13.54%, showing a low PCE loss compared to the small-area device. Our study paves the way towards the mass production of PSCs.

## 2. Materials and Methods

### 2.1. Materials

Chlorobenzene (CB, >99.0%), o-xylene (99%), anisole (99%), 1-pentanol (99%), N,N-dimethylformamide (DMF, 99.8%), dimethyl sulfoxide (DMSO, >99.9%), isopropyl alcohol (IPA, 99.8%), and polyethylene glycol (PEG) were purchased from Acros Organics, Geel, Belgium. Diethyl ether (99.0%) and ethyl alcohol (EtOH 99.99%) were obtained from Fisher Chemical, Hampton, NH, USA. 2-methylpyrazine (2-MP, >99%), polyethyleneimine (PEI, branched; MW = ca. 25,000), and tetrabutylammonium hydroxide 30-hydrate (TBAOH 30% H_2_O, 99%) were purchased from Sigma-Aldrich, St. Louis, MO, USA. FTO glasses, methylammonium iodide (MAI), lead iodide (PbI_2_, 99.9985%), and PC_61_BM (99.0%) were purchased from FrontMaterials Co., Ltd., Taoyuan, Taiwan. All the chemicals were used as received without any treatment.

### 2.2. Solution Preparations

For the hole transport layer (HTL), 124.40 mg nickel acetate tetrahydrate was dissolved in 1mL of ethanol, and then the solution was stirred at 60 °C until it became clear. After adding 30 µL of ethanolamine, the solution was filtered with 0.45 µm poly(1,1,2,2-tetrafluoroethylene) (PTFE). For slot-die coating, the solution of nickel acetate tetrahydrate with different concentrations were prepared in nitrogen for 1 day prior to use. The 42.70 wt% perovskite precursor solution was prepared for spin coating. For this purpose, 504 mg PbI_2_ and 176 mg MAI were dissolved in 240 μL of DMSO and 600 μL of DMF. The concentration of perovskite precursor solution for slot-die coating was reduced to 29.20 wt%, which contained 0.322 mg PbI_2_ and 113 mg MAI dissolved in the mixed solvent of DMSO:2MP. The PC_61_BM was used as the ETL, and the concentrations of PC_61_BM solution were 20 mg/mL in CB for spin coating and 15 mg/mL in o-xylene with anisole for slot-die coating, respectively. Two kinds of solutions of work function modifier were prepared in IPA or n-butanol, and the concentrations of work function modifier were 0.1 wt% of PEI in IPA for spin coating and TBAOH in n-butanol for slot-die coating.

### 2.3. Device Fabrication

For spin-coated PSCs, after washing a 2 × 2 cm FTO glasses sequentially with acetone, methanol, and isopropanol, the FTO glasses were treated with 15 min of O_2_ plasma treatment. The NiO_X_ solution was then spin-coated onto the clean FTO glasses at 4000 rpm for 20 s, followed by 20 min annealing in the air at 300 °C. Next, the NiO_X_/FTO samples were moved to the glove box and the perovskite precursor was spin-coated onto the NiO_X_ films at 4500 rpm for 30 s. At the 15th second, 300 μL of diethyl ether was dropped on the spin-coated perovskite film. The perovskite films were dried on the hot plate for 1 min at 75 °C and 2 min at 105 °C. To act as the ETL, the PC_61_BM solution was spin-coated onto the perovskite layer at 1000 rpm for 30 s. The PEI layer acted as the WF modifier layer and was deposited on PC_61_BM layer at 3000 rpm for 30 s. Finally, the Ag electrode was thermally evaporated on top of the PC_61_BM/PEI at a high vacuum (2 × 10^−6^ torr).

For the slot-die-coated PSCs, the HTL, perovskite layer, ETL, and WF layers were formed on the FTO substrate by using an automated slot-die machine (Coatema, Easy coater). The same FTO cleaning procedure as spin-coated PSCs was treated on 8 × 4 cm^2^ FTO glasses. The spacing between the slot-die head and the substrate was 200–220 μm for the NiO_X_ and perovskite specifications of the coating. The coating speed of 0.5 m/min and the feeding rate of 2.5 mL/min were used to regulate the wet film of the NiO_X_ precursor solution. The NiO_X_ film was then heated to 350 °C and annealed for 20 min after being maintained at 180 °C for 5 min on the hot plate. After annealing, the temperature was reduced by air cooling and the sample was then placed on a hot plate set to 170 °C for 5 min of preheating. Then, a wet film of the perovskite precursor solution was deposited on top of the NiO_X_ film at a coating speed of 3.0 m/min and a feeding rate of 2.0 mL/min. The ETL, PC_61_BM, was deposited on top of the perovskite layer at a coating speed of 3.5 m/min with a pump rate of 2.5 mL/min. The PEI, the WF modifier layer, was then coated on the PC_61_BM layer with a coating speed of 0.5 m/min and a feeding rate of 2.5 mL/min. Finally, Ag electrodes with a thickness of 100 nm were thermally evaporated on the PC_61_BM/PEI layer to complete the PSCs fabrication. For the fabrication of module, the procedure is the same as in our previous study [11]. In this study, we only connected two sub-cells in series, and the line widths of P1, P2, and P3 were 50 μm, 100 μm, and 100 μm, respectively.

### 2.4. Device Characterization

The surface morphology of the film has been studied by optical microscopy (Scope-A1, Zeiss, Jena, Germany), scanning electron microscopy (SEM, JSM-7610F, Jeol, Tokyo, Japan), and atomic force microscopy (AFM, OMV-NTSC, Bruker, Billerica, MA, USA). The crystal structures of perovskite films were characterized using an X-ray diffractometer (XRD, TTRAX iii, Rigaku, Tokyo, Japan). The steady-state photoluminescence (PL) spectra and time-resolved PL (TRPL) were performed by exciting perovskite with a 532 nm diode laser (LDH-P-C-405, PicoQuant, Berlin, Germany). The film thickness was examined using a 3D optical profiler (Contour Elite, Bruker, Billerica, MA, USA). The current–voltage curves of devices were measured by using a source meter (Keithley 2410) under 100 mW/cm^2^ illumination of AM 1.5 solar simulator (YSS-150A, Yamashita Denso, Tokyo, Japan). The external quantum efficiency (EQE) instrument (LSQE-R, LiveStrong Optoelectronics Co., Ltd., Kaohsiung, Taiwan) was used to measure the quantum efficiency of devices.

## 3. Results

### 3.1. Slot-Die Coating of NiO_X_ HTL

**Figure 1:**
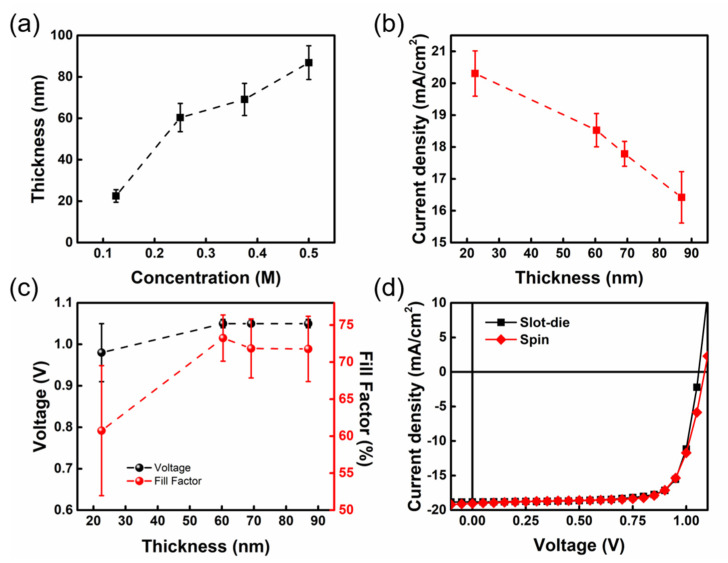
(**a**) Variation in the thickness of slot−die coated NiO_X_ films with molar concentration, (**b**) J_SC_, and (**c**) V_OC_ and FF of the PSCs with different thicknesses of slot-die coated NiO_X_ film. (**d**) J−V curves of PSCs fabricated from slot-die coated and spin-coated NiO_X_ film.

First, we investigated the effect of different precursor concentrations on the thickness of slot-die coated NiO_X_ thin films and the performance of PSCs. The fabrication of the three layers, namely the perovskite layer, PC_61_BM, and WF layer, was carried out by the spin coating process. The average thicknesses of NiO_X_ films fabricated at different precursor concentrations of 0.125 M, 0.25 M, 0.375 M, and 0.5 M are shown in Figure 1a. The average thicknesses with resistance and the device characteristics based on different NiO_X_ films are listed in Table S1. The results showed that the thickness of the NiO_X_ films became thicker with increasing NiO_X_ precursor concentration. The NiO_X_ film produced by 0.25 M has a thickness of 60.37 nm, which is quite similar to that of the spin-coated NiO_X_ film. As shown in Figure 1b, the short-circuit current density (J_SC_) of devices tends to decrease as the NiO_X_ film thickness increases. The J_SC_ value is impacted by the resistance (R), which increases with the long charge transport distance. The current–voltage (I-V) curves of the NiO_X_ films with various thicknesses can be used to determine the *R* relationship, as shown in Figure S1. Additionally, Figure 1c shows that the open-circuit voltage (V_OC_) and fill factor (FF) both increased considerably when the NiO_X_ thickness increased from 22.52 nm to 60.37 nm, rising from 1.01 V (66.22%) to 1.05 V (77.53%), respectively. The results show that different thicknesses of NiO_X_ film affect its coverage. In the SEM analysis shown in Figure S2, the FTO grains are stacked irregularly and the surface of FTO exhibits many protrusions. When only 22.52 nm of NiO_X_ was deposited on the FTO substrate, the incomplete coverage of NiO_X_ films made the perovskite layer directly contact with the FTO electrode, resulting in the loss of interfacial carriers. The underlying FTO substrate could be entirely covered by NiO_X_ film when the thickness of NiO_X_ was 60.37 nm, thereby improving the PCE of the PSCs. In addition, it can be observed from the SEM images that there is no significant difference in the surface morphology of NiO_X_ films fabricated from the two different coating techniques. The surface roughness of NiO_X_ films with various thicknesses were depicted in Figure S3 analyzed by AEM. The surface roughness (root-mean-square roughness, RMS) of pure FTO is 34.38 nm, and the deposition of 22.52 nm NiO_X_ cannot cover the FTO completely, which means that the RMS value only slightly decreases to 27.41 nm. As for the NiO_X_ film of 60.37 nm deposited on the FTO substrate, the surface roughness decreases significantly to 15.59 nm and becomes relatively flat. In this case, the PCE of the PSCs increases to 15.43%. This result indicates that the smooth NiO_X_ film facilitates the interfacial contact between the perovskite layer and the FTO electrode, thus, improving the PCE of the PSCs. Based on those results, the optimum concentration of the NiO_X_ HTL deposited by slot-die coating is 0.25 M. Figure 1d illustrates the J–V curves of slot-die coated and spin-coated PSCs, showing the similar PCEs of the PSCs fabricated by these two processes.

### 3.2. Slot-Die of Perovskite Films with Nontoxic Solvents

Perovskite precursors are commonly dissolved in polar solvents, such as DMF [28,29], GBL [30], and DMSO [31]. The most commonly used, DMF, is highly poisonous and volatile, making it unsuitable as the primary solvent for mass production of large-area perovskite solar cells. GBL and DMSO are less hazardous to the environment and have a high potential for usage in industry; the influence of GBL on human nerves is classified as a regulated drug in the European Union, and DMSO is a common industrial nontoxic solvent with no legal regulatory difficulties [32]. As a result, DMSO is an excellent choice as the primary solvent for the fabrication of large-area perovskite solar cells. However, DMSO exhibits a high boiling point (189 °C) and strong coordination with PbI_2_ [33], resulting in the formation of the perovskite films with a rough surface and pin-holes [30] and a lower PCE of PSCs. The combination of additional solvents with lower boiling points and surface tension has been described in the literature [34,35], and these methods improve wet film spreadability and boost solvent volatilization rate to achieve a very compact crystalline perovskite film. To lower the boiling point of the used solvent system, a nontoxic solvent with a low boiling point is used to construct a nontoxic solvent system with DMSO. The thickness is then tuned by modifying the process parameters and precursor concentration through the slot coating process. The surface morphology of the perovskite film can be optimized by modifying the temperature of the substrate and controlling the volatilization rate of the solvent system. It has been demonstrated that the low boiling point nontoxic solvent 2-MP can mix and dissolve the perovskite precursor uniformly with DMSO to obtain a high PCE [36]. 2-MP not only reduces the harm to humans, but also weakens the interaction between DMSO and PbI_2_ due to its good miscibility with DMSO. In addition, the high saturation vapor pressure of 2-MP allows it to be rapidly removed from the perovskite crystals during the film formation process. These reasons make us obtain high quality perovskite films using 2-MP as the perovskite precursor host solvent. Therefore, we used the mixing solvents of DMSO and 2-MP as the host solvent of the perovskite precursor for the slot-die coated PSCs.

Table S2 lists the physical parameters of several solvents, including vapor pressure, surface tension at room temperature, and boiling point. The higher the vapor pressure at room temperature, the easier it is for the solvent to change from a liquid to a gaseous state. Therefore, when the solvent is removed by heating, it volatilizes faster and a uniform film surface is achieved. The lower the surface tension, the easier it is to spread evenly on the substrate surface after coating. The surface tension of DMSO before mixing decreased from 37.81 mN/m to 29.65 mN/m, and the vapor pressure of 2-MP at room temperature was 1292 Pa, which is much higher than that of DMSO at room temperature of 56 Pa, so mixing DMSO with 2-MP can improve the volatility of DMSO. When 2-MP, a low boiling point solvent, is mixed with DMSO, the high surface tension of DMSO is successfully reduced, which is beneficial to the coating layer.

**Figure 2:**
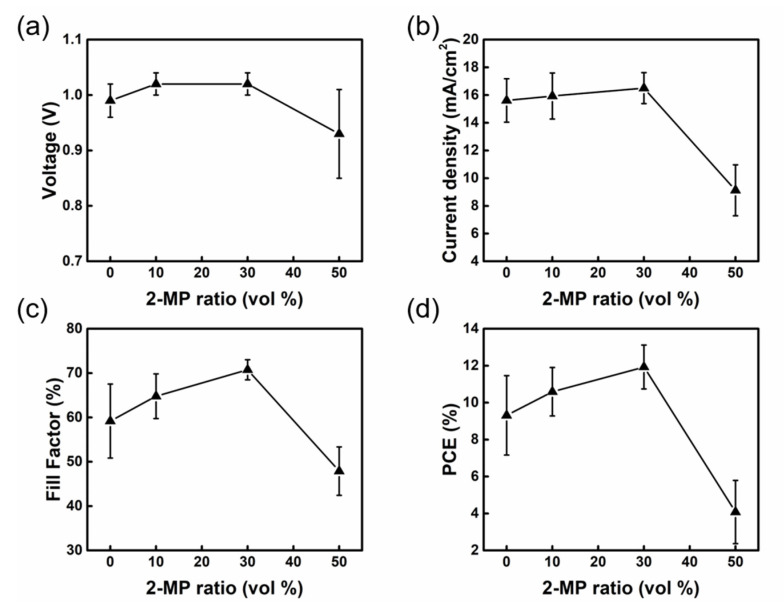
Variation in photovoltaic characteristics of slot-die coated PSCs fabricated with different 2-MP ratios. (**a**) V_OC_, (**b**) J_SC_, (**c**) FF, and (**d**) PCE.

We discussed the effect of the solvent mixture ratio on the device performance, and the results in Figure 2a–d show that the PCE is only 9.31% when using pure DMSO (10:0). When DMSO is mixed with 2-MP in 9:1 ratio, J_SC_ increases from 15.61 mA/cm^2^ to 15.93 mA/cm^2^, V_OC_ increases from 0.99 V to 1.02 V, and FF increases from 59.18% to 64.79%, resulting in an enhanced PCE of 10.59%. When the mixing ratio of 2-MP to DMSO was increased to 7:3, the V_OC_ remained at 1.02 V and the J_SC_ and FF continued to rise, resulting in an improvement in PCE to 11.93%. However, it can be observed that J_SC_, V_OC_, and FF decreased when the raising mixing ratio of 2-MP approached 50%, and the PCE dramatically reduced to 4.08%. It has been discovered that the perovskite precursor can be well blended with the three solvent mixing ratios of 10:0, 9:1, and 7:3 to produce a transparent and clean solution without aggregates. When the solvent mixing ratio is 5:5, the perovskite precursor solution becomes murky as a result of the excessive ratio of 2-MP with the low solubility of PbI_2_ in the mixed solvent system. The PbI_2_ aggregates dispersed on the perovskite film would affect the shunt resistance of the device and result in low PCE. The PCE increases with the mixing ratio of 2-MP in the mixed solvent in the case of a clear perovskite precursor solution. As the amount of 2-MP in the solvent system rises, the produced perovskite wet film becomes more volatile, has lower surface tension, and does not form a complex with PbI_2_ compared to the use of pure DMSO. Consequently, 7:3 is the ideal mixing ratio of DMSO to 2-MP solvents for the slot coating process of PSCs. Table 1 lists comprehensive device photovoltaic characteristics.

**Figure 3:**
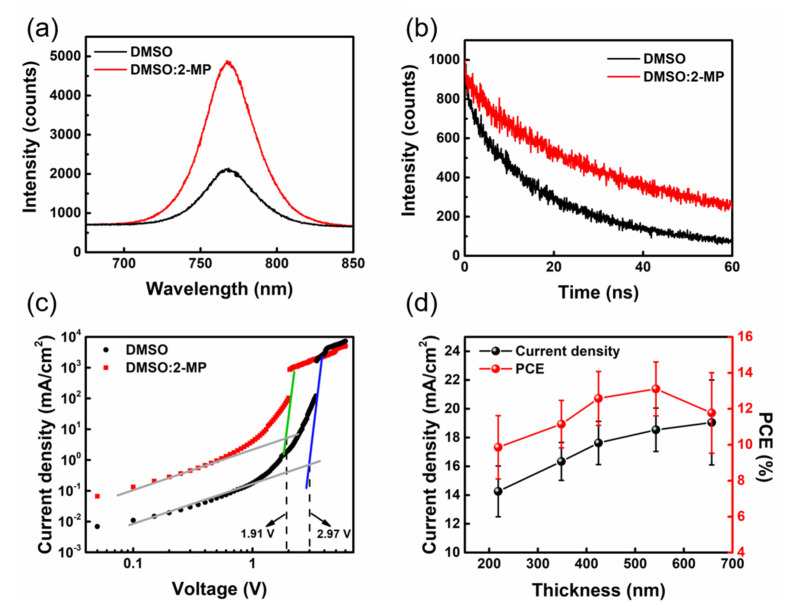
Comparison of slot−die coated perovskite films prepared from DMSO and DMSO:2−MP (70:30 vol%). (**a**) PL spectrum, (**b**) TRPL kinetics, (**c**) SCLC curve, and (**d**) variation in current density and PCE according to the thickness of slot-die coated perovskite films.

To understand the qualitative differences between the perovskite films fabricated from various solvent systems, the optical properties and carrier behavior were analyzed by PL and TRPL. Figure 3a illustrates that the PL intensity of the perovskite film made from the DMSO:2-MP solvent system is higher than that of the film from pure DMSO, indicating that the perovskite films made from the DMSO:2-MP solvent system have better film quality and fewer defects [37]. The TRPL spectra of these perovskite thin films shown in Figure 3b are fitted by a biexponential decay function, and the fitting results are shown in Table S3. Compared to the film fabricated from DMSO solvent, the average lifetime of the perovskite film fabricated from DMSO:2-MP solvent increases from 16.73 ns to 32.64 ns, implying that the number of defects in the film was effectively reduced and, therefore, lowering the possibility of carrier recombination. The space-charge-limited current (SCLC) model was also used to investigate the difference in charge defect density of these perovskite films. In Figure 3c, the J-V curve of the perovskite films made using the two solvents were measured. These films have a single-carrier hole element structure. The trap filled limited voltage (V_TFL_) of the films fabricated from DMSO and DMSO:2-MP are 2.97 V and 1.91 V, respectively. The values of charge trap density of the two perovskite films are 3.33 × 10^−16^ cm^−3^ (DMSO system) and 5.18 × 10^−16^ cm^−3^ (DMSO:2-MP system), respectively. The perovskite films fabricated from DMSO:2-MP exhibit a lower charge trap density, indicating less recombination and enhancing the PCE of perovskite solar cells. In addition, film thickness has a significant impact on the performance of perovskite solar cells, and it can be adjusted by tuning the precursor concentration and other processing parameters. As the thickness rises, it is expected that the absorption of sunlight by the active layer will increase in order to enhance the current density and produce more electron–hole pairs [38]. Figure 3d shows the variation in current density and PCE of the PSCs for different thicknesses of the slot-die coated perovskite films, and the detailed photovoltaics characteristics are listed in Table S4. According to the results, the PCE of the devices is 11.44% and the J_SC_ is only 14.86 mA/cm^2^ when the perovskite film thickness is 218.47 nm. While there is no significant change in V_OC_ and FF as the thickness reaches 542.63 nm, the J_SC_ rises to 18.98 mA/cm^2^, resulting in an optimal PCE of 14.13%. As the film thickness increased from 542.63 nm to 657.19 nm, although the resulting J_SC_ increased from 18.98 mA/cm^2^ to 19.42 mA/cm^2^, the FF reduced from 70.47% to 63.19% and the PCE of devices decreased from 14.13% to 13.07%. We examined the external quantum efficiency (EQE) spectrum of the above-mentioned perovskite solar cells with various thicknesses to verify the J_SC_ trend. Figure S4 shows that the EQE curves move upward with the increasing thickness of perovskite films, increasing the integrated area under the line and the simulated current density of PSCs, which is consistent with the measured current density trend. In addition to perovskite film thickness, the film morphology and surface roughness are also important factors for the PCE of slot-die coated PSCs. The AFM analysis was used to investigate the roughness of the perovskite films with different thicknesses and to better understand the longitudinal information of the film surface. Figure S5 depicts the AFM image of perovskite films with different thicknesses. The perovskite film with a thickness of 542.63 nm displayed a uniform surface without obvious height differences (with an RMS value of 42.17 nm), and the perovskite active layer would be in direct contact with the electrode. As the film thickness approached 657.19 nm, the surface of the perovskite film became uneven and clearly depressed, resulting in an increased RMS value of 91.86 nm. As a result, the FF value of the perovskite film with a thickness of 657.19 nm significantly decreased. A high RMS value of the film surface would adversely affect the succeeding PC_61_BM deposition; therefore, the ideal thickness for slot-die coated perovskite films is about 542.63 nm.

**Figure 4:**
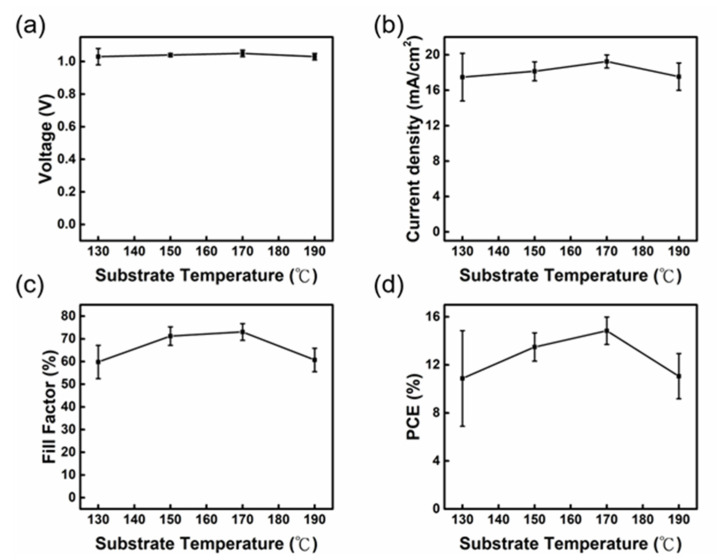
Variation in (**a**) V_OC_, (**b**) J_SC_, (**c**) FF, and (**d**) PCE of the slot-die coated PSCs fabricated at different preheated FTO/NiO_X_ substrate temperature.

The slot-die coating process involves the immediate volatilization of solvent during the perovskite film formation, causing the rapid formation of the perovskite crystal. The efficiency of the planar structure perovskite solar cell is influenced by the surface morphology and crystallinity of the perovskite thin film. Because we used the slot-die coating method to deposit the perovskite film directly on the preheated FTO/NiO_X_ substrate, we have to examine the effect of substrate temperature on the device performance. Figure 4a–d displays the trend of devices’ photovoltaic characteristics operated at various substrate temperatures, and the detailed values are listed in Table S5. When the substrate temperature is below 170 °C, the solvent cannot volatilize quickly, resulting in incomplete crystallization of the perovskite film and poor J_SC_ and FF. The J_SC_ and FF reach their maximum at the substrate temperature of 170 °C. When the substrate temperature is raised to 190 °C, which is over the boiling point of DMSO, the solvent quickly volatilizes and generates an irregularly arranged perovskite crystal with many cracks. XRD data is shown in Figure S6. The diffraction angles at 14.1°, 19.8°, 28.5°, and 40.5° are the Bragg diffraction peaks corresponding to the (110), (112), (220), and (224) planes of the methylamino lead iodide (MAPbI_3_) perovskite crystal. When the substrate temperature is below 170 °C, the perovskite film has no crystallographic orientation. When the substrate temperature is set to 170 °C, the diffraction intensity of the (112) plane is significantly increased, whereas the intensity of the (110) plane is decreased. This phenomenon illustrates that the crystal prefers to grow in the direction perpendicular to the (112) plane. The preferential crystal orientation would make the perovskite crystal stacked more tightly with fewer grain boundaries, resulting in better photovoltaic properties [39].

**Figure 5:**
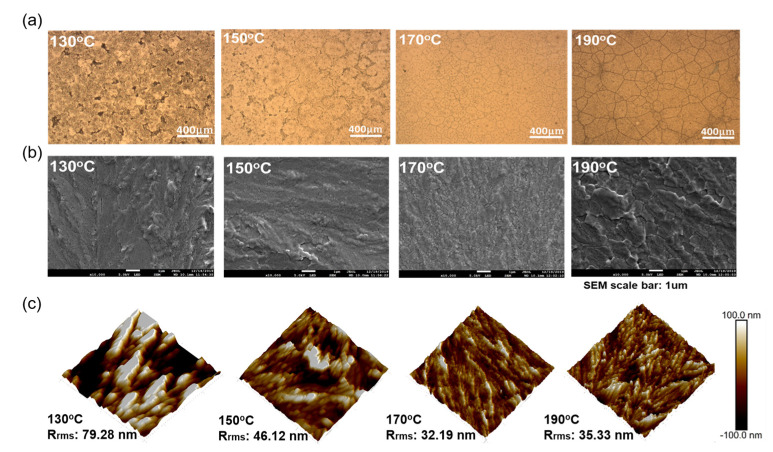
Perovskite film with different preheated FTO/NiO_X_ substrate temperatures. (**a**) OM image, (**b**) top view SEM image, and (**c**) AFM image.

The variation in the surface morphology of slot-die coated perovskite films at different substrate temperatures is also discussed. From the OM images shown in Figure 5a, the perovskite film with a substrate temperature below 170 °C easily forms a large number of pinholes, while at 190 °C the film shows many cracks, which is consistent with our assumption above. From the SEM images shown in Figure 5b, it can be observed that the perovskite crystals will stack on top of each other in an irregular shape when the solvent evaporates too slowly. As the substrate temperature is increased to 170 °C, the thermal energy is sufficient to remove the solvent uniformly; thus, the uniform and flat perovskite film can be obtained. When further heating the substrate to a temperature higher than the boiling point of the solvent, although larger perovskite grains can be formed, the arising of the grain boundaries resulted in poor carrier transport and device performance. The surface morphology of the perovskite film deposited at different substrate temperatures has also been investigated by AFM images, as shown in Figure 5c. At a low substrate temperature of 130 °C, the film surface is not well covered with dense pores, resulting in an island-like, uneven surface profile with an RMS value of 79.28 nm. The rough surface confines the transportation of carriers. When the substrate temperature is raised to 170 °C, the pores in the film are reduced. The grains are arranged more densely, the surface becomes flatter, and the RMS value decreases to 32.19 nm. Cracks appear on the surface of the film when the substrate temperature continues to be increased to 190 °C, and the RMS value increases to 35.33 nm. From the above detailed analysis of the results, the ideal substrate temperature for the slot-die-coated perovskite film is 170 °C.

### 3.3. Slot-Die Coating of PC_61_BM and WF Layer Using Nontoxic Solvent

PC_61_BM is employed as the ETL of PSCs in this work. Within the recent literature, CB or dichlorobenzene (DCB) are commonly used as solvents for PC_61_BM [40,41]. However, the halide groups make these solvents possess bio-toxicity and need to be replaced for the mass production process. Here, we choose the o-xylene and anisole as nontoxic solvents for PC_61_BM. Table S6 listed the characteristics of PSCs with PC_61_BM ETL made by o-xylene and anisole for the slot-die coating and CB for the spin coating processes. The results demonstrate that when o-xylene is used as the solvent, the values of J_SC_, V_OC_, FF of the PSCs are all close to the average value as using CB as solvent in spin coating process, and the average PCE is 11.41%. On the other hand, the V_OC_ and FF of the PSCs significantly decrease when anisole is used, and the average PCE is only 8.64%. The difference in surface tension between o-xylene and anisole over the perovskite film affects the coverage of PC_61_BM ETL.

Figure S7 displays AFM images of PC_61_BM films in various solvents. It has been noted that when o-xylene is employed as a solvent, it can be uniformly spread over the surface of perovskite film during slot-die coating and can completely cover the surface of perovskite film after deposition and drying. The RMS value of the initial perovskite layer was approximately 32.19 nm; after coating the o-xylene PC_61_BM ETL, the RMS value decreased to 25.24 nm. Furthermore, when anisole is employed as the solvent, the spreading effect of the solution is poor because of the high surface tension, and many pores appeared on the surface after deposition and drying, causing the RMS value rise from 32.19 nm to 45.17 nm. Therefore, we utilized o-xylene as a substitute solvent for mass production. The thickness of PC_61_BM ETL is unaffected by adjusting the solution concentration, since it employs o-xylene, which is less soluble than CB.

**Figure 6:**
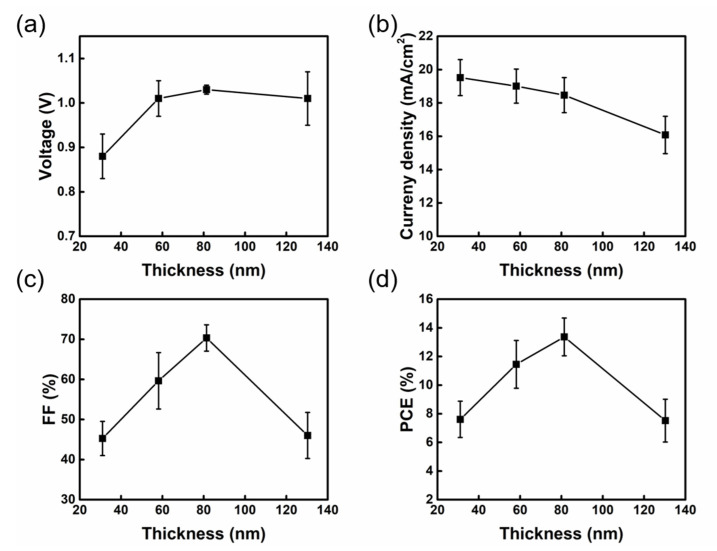
Variation in thickness with different parameters of (**a**) V_OC_, (**b**) J_SC_, (**c**) FF, and (**d**) PCE of the PSCs fabricated with different PC_61_BM thicknesses.

It is also important to adjust the slot-die coating rate to optimize the thickness of PC_61_BM ETL until it completely covers the perovskite layer. The PC_61_BM thickness was changed with various coating rates of 1.5 m/min, 2.5 m/min, 3.5 m/min, and 4.5 m/min to obtain the ideal film thickness in comparison to the spin coating procedure. The respective thicknesses are 31.03, 58.14, 81.38, and 130.28 nm. Figure 6a–d shows the characteristics of PSCs with various PC_61_BM thicknesses and Table 2 summarizes the performances of the device. The J_SC_ of the device tends to decrease with the raising thickness of PC_61_BM film. The primary reason is that as PC_61_BM thickness increases, charge transport distance also increases. The increase in the possibility of a charge encountering the defect along the transport pathway may cause an increasing series resistance [42], which in turn influences the J_SC_ value. As a result, the resistance of the PC_61_BM film is measured to examine how thickness affects electrical attributes. In accordance with Ohm’s law, the resistance increases while the slope of the J-V straight line exhibits a decreasing trend, which is consistent with the growth of PC_61_BM thickness, as shown in Figure S8. Although the maximum J_SC_ and the lowest resistance value can be achieved when the PC_61_BM thickness is 31.03 nm, the low V_OC_ and FF (0.94 V and 49.81%, respectively) restricted the PCE of PSCs. V_OC_ and FF presented a rising trend when PC_61_BM thickness grew from 31.03 nm to 81.38 nm, and achieved the maximum average PCE of 13.37% when the PC_61_BM thickness was 81.38 nm.

**Figure 7:**
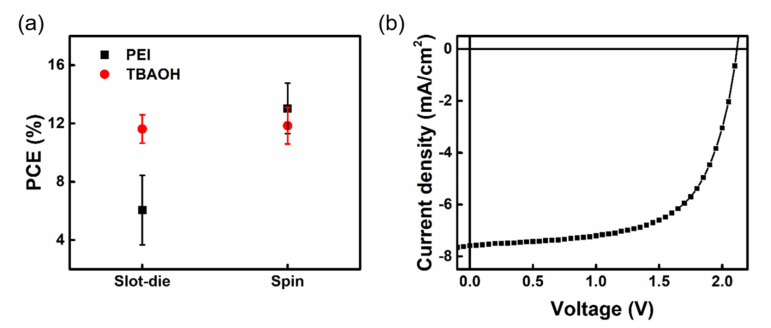
(**a**) Distribution of power conversion efficiencies of PSCs fabricated with different WF modified materials prepared by slot−die coating and spin coating. (**b**) J−V curve of mini-module PSCs under a device area of 0.75 cm^2^.

Figure S9 displays the OM image of spin-coated, PC_61_BM, and slot-die coated PC_61_BM on perovskite film at various thicknesses. When 31.03 nm of PC_61_BM was deposited on the perovskite film, the underlying perovskite was essentially not covered, leading to the direct contact of the silver electrodes. Due to the poor carrier conduction in the interface, the V_OC_ and FF are quite low. When the PC_61_BM thickness increases to 58.14 nm, the coverage of the perovskite increases compared with the deposition of 31.03 nm PC_61_BM. The V_OC_ and FF values increase with the increase in the coverage; however, the PCE of the device is still not as good as that of spin coating. When the PC_61_BM thickness increased to 81.38 nm, the PC_61_BM could evenly and completely cover the underlying perovskite. Its surface morphology is similar to the PC_61_BM film prepared by the standard spin coating process, and, therefore, their device performances are close to each other. When the PC_61_BM thickness is further increased to 130.28 nm, it can be seen that the color of the PC_61_BM film becomes darker. Despite the coverage of the perovskite being quite good, the thick PC_61_BM film makes it easy for the carriers to recombine before transporting to the top electrode [43], resulting in a significant decrease in J_SC_ and FF values. In the part of the WF layer, the commonly used PEI in the spin coating process contains tertiary amines with lone pairs of electrons. When the wet PEI film of the slot-die coating needs to stand on the substrate for a relatively longer time than spin coating, it may destroy the perovskite structure and is, thus, not suitable for slot-die coating, as shown in Table S7. Here, we use TBAOH as a substitute WF layer, which has an interface modification function. The TBAOH is composed of quaternary amines, and the nitrogen does not have lone pair electrons to react with MAI or destroy the perovskite structure. According to our previous reports [44], TBAOH can improve the work function of silver. Comparing PEI and TBAOH in different process technologies, TBAOH shows a larger process window, as shown in Figure 7a. Finally, we made a module with an effective area of 0.75 cm^2^. Figure S10 shows the schematic diagram of the module, where P1 was cut by laser and P2 and P3 were made with tweezers. The parameters of the deposition of all the layers for the module are the same as the procedure for slot-die-coated PSCs. The energy band diagram of the devices is shown in Figure S11. Its overall module J-V is shown in Figure 7b, and the PCE reaches 13.54%, which is similar to that of the device fabricated from spin coating, as shown in Table S8. In conclusion, we have successfully fabricated PSCs by using the slot-die process with nontoxic solvents under ambient condition. This work shows the high potential for commercial mass production of PSCs.

## 4. Conclusions

In this study, an environmentally friendly mass production process of PSCs was successfully established. Except for the top electrode, each layer of the device was deposited by the slot-die coating method with nontoxic solvents. The PSCs were prepared in the p-i-n device structure (FTO/NiO_X_/MAPbI_3_/PC_61_BM/TBAOH/Ag). The influencing factors of each layer on the mass production process of PSCs were studied with its improvement mechanism. Using this process system, a module with an effective area of 0.75 cm^2^ shows a PCE of 13.54%, which is similar to that of the devices with a small-area (0.09 cm^2^ active area) cell. This work demonstrates the potential for mass production PSCs in the future with our established process.

## Figures and Tables

**Table 1:** Performance of PSCs fabricated using DMSO:2-MP mixing solvents with different 2-MP ratios in ambient condition. The data were averaged from 20 devices.

2-MP. (Vol%)	V_OC_ (V)	J_SC_ (mA/cm^2^)	FF (%)	PCE (%)
0	0.99 ± 0.03	15.61 ± 1.57	59.18 ± 8.36	9.31 ± 2.15
10	1.02 ± 0.02	15.93 ± 1.66	64.79 ± 5.04	10.59 ± 1.31
30	1.02 ± 0.02	16.50 ± 1.12	70.75 ± 2.27	11.93 ± 1.19
50	0.93 ± 0.08	9.13 ± 1.84	47.89 ± 5.45	4.08 ± 1.71

**Table 2:** Device performance of PSCs with different PC_61_BM thicknesses. The data were averaged from 20 devices.

Process	Moving Rate (m/min)	Thickness (nm)	R (10^−1^ ohm)	V_OC_ (V)	J_SC_ (mA/cm^2^)	FF (%)	PCE (%)
Slot−die (SD)	1.5	31.03 ± 7.61	9.30	0.88 ± 0.05 (0.94)	19.52 ± 1.08 (20.98)	45.27 ± 4.25 (49.81)	7.61 ± 1.27 (9.88)
	2.5	58.14 ± 7.52	12.58	1.01 ± 0.04 (1.03)	19.01 ± 1.02 (18.76)	59.65 ± 7.04 (68.99)	11.45 ± 1.67 (13.46)
	3.5	81.38 ± 8.09	18.58	1.03 ± 0.01 (1.07)	18.47 ± 1.05 (19.18)	70.33 ± 3.28 (77.17)	13.37 ± 1.32 (15.95)
	4.5	130.28 ± 29.06	29.47	1.01 ± 0.06 (1.04)	16.08 ± 1.12 (18.29)	46.02 ± 5.75 (57.30)	7.52 ± 1.49 (10.98)
Spin (SP)	-	80.44 ± 5.01	18.33	1.03 ± 0.02 (1.06)	18.44 ± 0.80 (19.34)	71.52 ± 3.71 (74.72)	13.68 ± 1.09 (15.35)

## Data Availability

Data are available from the corresponding author upon reasonable request.

## Supplementary Materials

The following supporting information can be downloaded at: https://www.mdpi.com/article/10.3390/nano13111760/s1, Table S1. The thickness, resistance (R), and device performance of slot-die coated PSCs with different thickness of NiO_X_ film compared with spin-coated PSCs; Table S2. Physical properties of solvents; Table S3. Fitting results of time-resolved photoluminescence characterization; Table S4. Device performance of different thicknesses of perovskite film by slot-die coating; Table S5. Device performance of slot-die coated PSCs with perovskite film fabricated at different substrate temperatures; Table S6. Device performance of slot-die coated PSCs using PC_61_BM films fabricated by different solvents compared with spin-coated PSCs; Table S7. Device performance of slot-die coated PSCs with different WF materials compared with spin-coated PSCs; Table S8. Device performance of standard perovskite solar cell and 4 cm^2^ substrate area module. The J_SC_ and PCE are performed under active area; Figure S1. The I-V curve of slot-die coated NiO_X_ film with different thicknesses and spin-coated NiO_X_ film obtained by linear sweep voltammetry; Figure S2. Top view SEM image of FTO, 0.125 M and 0.25 M NiO_X_ film by slot-die, 0.5 M NiO_X_ film by spin coating; Figure S3. AFM image of FTO, 0.125 M and 0.25 M NiO_X_ film by slot-die, 0.5 M NiO_X_ film by spin coating; Figure S4. EQE spectrum with different thicknesses of perovskite film by slot-die coating; Figure S5. Different thicknesses of slot-die perovskite film: (a) 542.63 nm, (b) 657.19 nm; Figure S6. XRD patterns of perovskite films fabricated at different substrate temperature; Figure S7. AFM image of PC_61_BM using anisole and o-xylene solvent; Figure S8. The I-V curve of slot-die coated PC_61_BM film with different moving rates and spin-coated PC_61_BM film obtained by linear sweep voltammetry; Figure S9. OM images of slot-die coated PC_61_BM film with different thicknesses and spin-coated PC_61_BM film; Figure S10. The schematic diagram of the module. The line widths of P1, P2, and P3 were 50 μm, 100 μm, and 100 μm, respectively; Figure S11. The energy band diagram of the perovskite device.
