# Supplementary material for: High-Performance Perovskite Solar Cells and Modules Fabricated by Slot-Die Coating with Nontoxic Solvents
Source: Nanomaterials (Basel). 2023 May 29;13(11):1760. doi: 10.3390/nano13111760 (PMC10254507; doi:10.3390/nano13111760)
Supplement: Supplementary file 1 [file nanomaterials-13-01760-s001.zip › nanomaterials-2370117-supplementary.pdf]

Supporting information

High-Performance Perovskite Solar Cells and Modules Fabricated by Slot-Die Coating with Nontoxic Solvents

Chia-Feng Li [1,2], Hung-Che Huang [1], Shih-Han Huang [2], Yu-Hung Hsiao [2], Priyanka Chaudhary [2], Chun-Yu Chang [3], Feng-Yu Tsai [1], Wei-Fang Su [1,2] and Yu-Ching Huang [2,4,5,*]

[1] Department of Materials Science and Engineering, National Taiwan University, Taipei 10617, Taiwan; d10527005@ntu.edu.tw (C.-F.L.); r06527061@ntu.edu.tw (H.-C.H.); ftsai@ntu.edu.tw (F.-Y.T.); suwf@ntu.edu.tw (W.-F.S.)
[2] Department of Materials Engineering, Ming Chi University of Technology, New Taipei City 24301, Taiwan; d05527006@ntu.edu.tw (S.-H.H.); u08187148@mail2.mcut.edu.tw (Y.-H.H.);
priyanka@mail.mcut.edu.tw (P.C.)
[3] Sumei Chemical Co., Ltd., Tao Yuan City 32849, Taiwan; october32019@gmail.com (C.-Y.C.)
[4] Organic Electronics Research Center, Ming Chi University of Technology, New Taipei City 24301, Taiwan
[5] Center for Plasma and Thin Film Technologies, Ming Chi University of Technology, New Taipei City 24301, Taiwan
* Correspondence: huangyc@mail.mcut.edu.tw; Tel.: +886-2-2908-9899 (ext. 4477)

Table S1. The thickness, resistance (R) and device performance of slot-die coated PSCs with different precursor concentration of NiO_x film compared with spin coated PSCs.

Process	Precursor concentration (M)	Thickness (nm)	R (10^{-1}ohm)	V_{OC} (V)	J_{SC} (mA/cm^2)	FF (%)	PCE (%)
Slot-die (SD)	0.125	22.52 ±3.03	8.04	0.98 ±0.07 (1.01)	20.30±0.71 (19.46)	60.74 ±8.78 (66.22)	12.26 ±2.23 (13.12)
	0.25	60.37 ±6.84	12.44	1.05 ±0.01 (1.05)	18.52 ±0.52 (18.80)	73.24 ±3.12 (77.53)	14.30 ±0.81 (15.43)
	0.375	69.12±7.76	14.65	1.05 ±0.00 (1.06)	17.78 ±0.38 (17.45)	71.84 ±3.98 (77.17)	13.47 ±0.77 (14.30)
	0.50	86.88±8.15	19.05	1.05 ±0.01 (1.06)	16.41 ±0.80 (17.06)	71.78 ±4.41 (74.85)	12.47 ±1.07 (13.57)

Formatted: Font: Not Bold
Formatted: Font: Not Bold
Formatted: Font: Not Bold
Formatted: Font: Not Bold
Formatted: Font: Not Bold
Formatted: Font: Not Bold
Formatted: Font: Not Bold
Formatted: Font: Not Bold
Formatted: Font: Not Bold
Formatted: Font: Not Bold
Formatted: Font: Not Bold
Formatted: Font: Not Bold
Formatted: Font: Not Bold
Formatted: Font: Not Bold
Formatted: Font: Not Bold
Formatted: Font: Not Bold
Formatted: Font: Not Bold

Spin (SP)	0.50	61.30 ±6.59	12.64	1.05 ±0.01 (1.08)	18.31 ±0.72 (19.06)	73.98 ±2.33 (74.46)	14.28 ±0.52 (15.41)

Formatted: Font: Not Bold
Formatted: Font: Not Bold
Formatted: Font: Not Bold
Formatted: Font: Not Bold
Formatted: Font: Not Bold

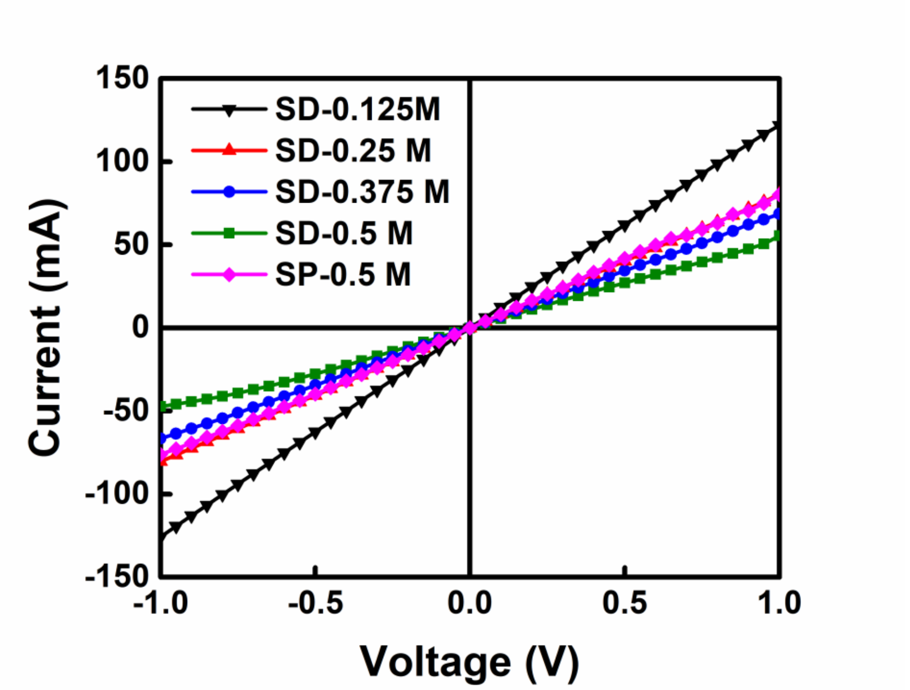

Figure S1. The I–V curve of slot-die coated NiO_x ~~x~~ film with precursor concentration and spin coated ~~NiOx~~ NiO_x film obtained by linear sweep voltammetry.

Formatted: Subscript
Formatted: Subscript

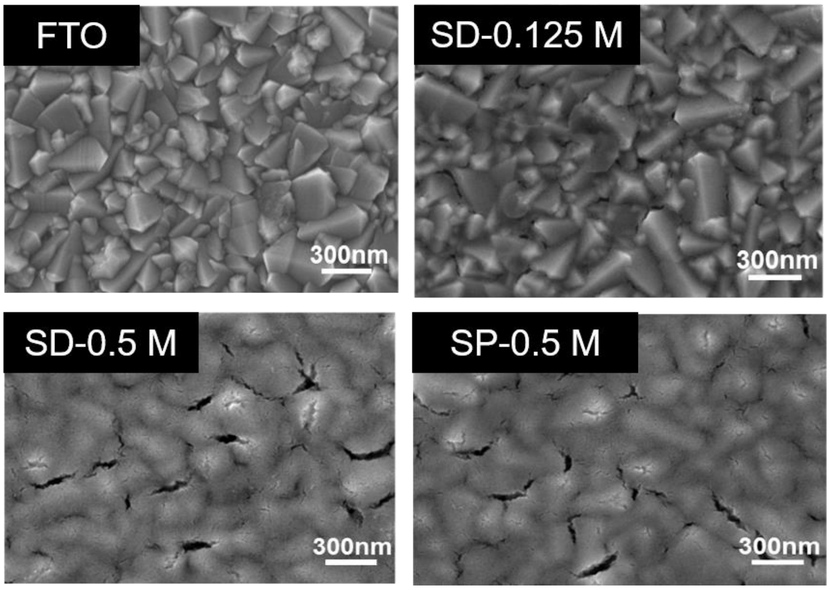

Figure S2. Top view SEM image of FTO, 0.125 M and 0.25 M nm NiO_x film by slot-die, 0.5 M NiO_x film by spin coating.

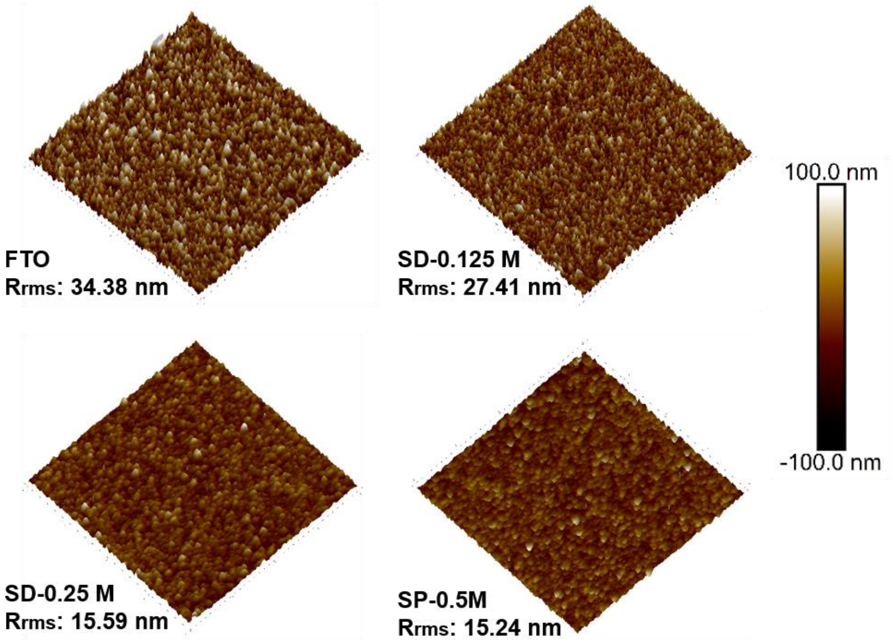

Figure S3. AFM image of FTO, 0.125 M and 0.25 M NiO_x film by slot-die, 0.5 M NiO_x film by spin coating.

Table S2. Physical properties of solvents.

**Solvent**	**Vapor pressure at R.T. (Pa)**	**Surface tension (mN m^{-1})**	**Boiling point (°C)**
DMF	516	33.78	152
DMSO	56	37.81	189
2-MP	1292	26.23	135
DMSO+2-MP	-	29.65	-

Table S3. Fitting results of time-resolved photoluminescence characterization.

**Solvent**	**τ_1 (ns)**	**Fraction (%)**	**τ_2 (ns)**	**Fraction (%)**	**$\tau_{average}$ (ns)**
DMSO	21.54	75	2.27	25	16.73
DMSO:2-MP (30 vol%)	36.59	88	3.72	12	32.64

Table S4. Device performance of different thickness of perovskite film by slot-die coating.

**Thickness (nm)**	**V_{OC} (V)**	**J_{SC} (mA/cm^2)**	**FF (%)**	**PCE (%)**
218.47±29.21	1.03 ±0.02 (1.05)	14.26 ±1.87 (14.86)	67.16 ±5.32 (72.79)	9.86 ±1.76 (11.44)

Formatted: Font: Not Bold

Formatted: Font: Not Bold

Formatted: Font: Not Bold

Formatted: Font: Not Bold

348.28±34.86	1.01 ±0.01 (1.01)	16.34 ±1.03 (17.18)	67.58 ±4.48 (71.32)	11.15 ±1.32 (12.46)
424.55±36.56	1.02 ±0.02 (1.03)	17.63 ±1.33 (18.19)	69.73 ±3.83 (71.63)	12.58 ±1.50 (13.48)
542.63±28.25	1.03 ±0.02 (1.05)	18.54 ±1.02 (18.98)	68.13 ±5.23 (70.47)	13.11 ±1.50 (14.13)
657.19±62.36	1.03 ±0.04 (1.06)	19.05 ±2.95 (19.42)	59.53 ±7.69 (63.19)	11.77 ±2.24 (13.07)

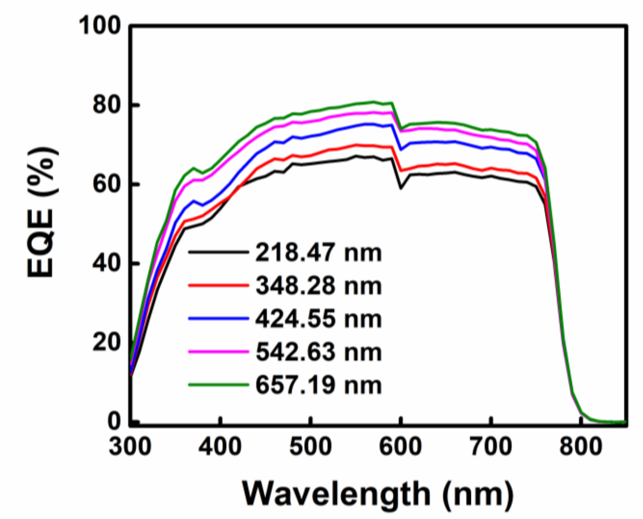

Figure S4. EQE spectrum with different thickness of perovskite film by slot–-die coating.

Formatted: Font: Not Bold (×16)

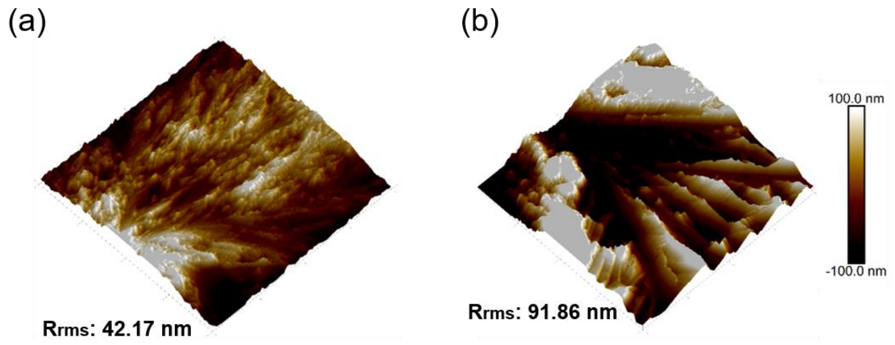

Figure S5. Different thickness of slot-die perovskite film (a) 542.63 nm, (b)657.19 nm.

Table S5. Device performance of slot-die coated PSCs with perovskite film fabricated at different substrate temperature.

**Substrate temperature** (℃)	V_{OC} **(V)**	J_{SC} **(mA/cm²)**	**FF (%)**	**PCE (%)**
130	1.03 ±0.05 (1.05)	17.48 ±2.69 (18.28)	59.82 ±7.34 (64.82)	10.87 ±3.98 (12.51)
150	1.04 ±0.01 (1.05)	18.14 ±1.07 (18.24)	71.22 ±4.08 (73.31)	13.49 ±1.17 (14.11)
170	1.05 ±0.02 (1.06)	19.25 ±0.73 (19.88)	73.05 ±3.65 (76.22)	14.84 ±1.14 (16.09)
190	1.03 ±0.02 (1.06)	17.53 ±1.54 (18.66)	60.71 ±5.17 (66.54)	11.06 ±1.88 (13.16)

Formatted: Font: Not Bold
Formatted: Font: Not Bold
Formatted: Font: Not Bold
Formatted: Font: Not Bold
Formatted: Font: Not Bold
Formatted: Font: Not Bold
Formatted: Font: Not Bold
Formatted: Font: Not Bold
Formatted: Font: Not Bold
Formatted: Font: Not Bold
Formatted: Font: Not Bold
Formatted: Font: Not Bold
Formatted: Font: Not Bold
Formatted: Font: Not Bold
Formatted: Font: Not Bold
Formatted: Font: Not Bold

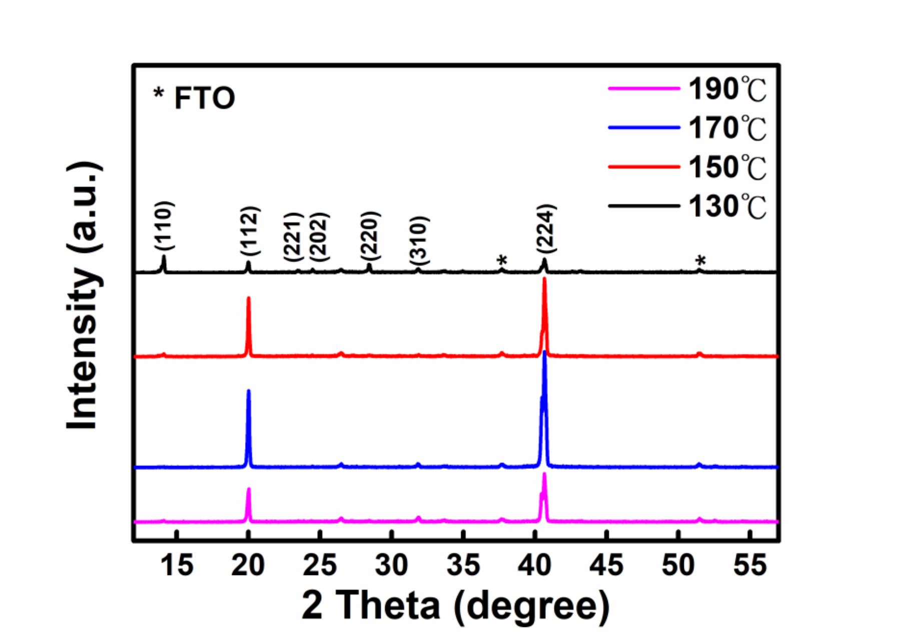

Figure S6. XRD patterns of perovskite films fabricated at different substrate temperature.

Table S6. Device performance of slot-die coated PSCs using $PC_{61}BM$ films fabricated by different solvent compared with spin coated PSCs.

**Process**	**PCBM solvent**	**V_{OC} (V)**	**J_{SC} (mA/cm^2)**	**FF (%)**	**PCE (%)**
Slot-die (SD)	o-xylene	1.02 ±0.02 (1.06)	17.31 ±1.16 (19.35)	64.10 ±4.88 (66.65)	11.41 ±1.24 (13.64)
	anisole	0.95 ±0.05 (1.01)	17.08 ±1.83 (19.25)	51.62 ±8.45 (63.03)	8.64 ±2.37 (12.36)
	CB	1.02 ±0.01 (1.02)	17.49 ±0.88 (18.10)	64.50 ±5.10 (73.52)	11.52 ±1.01 (13.62)

Formatted: Font: Not Bold
Formatted: Font: Not Bold
Formatted: Font: Not Bold
Formatted: Font: Not Bold
Formatted: Font: Not Bold
Formatted: Font: Not Bold
Formatted: Font: Not Bold
Formatted: Font: Not Bold
Formatted: Font: Not Bold
Formatted: Font: Not Bold
Formatted: Font: Not Bold
Formatted: Font: Not Bold

Spin (SP)	CB	1.03 ±0.02 (1.06)	18.66 ±0.63 (19.34)	71.88 ±3.67 (74.72)	13.86 ±0.92 (15.35)

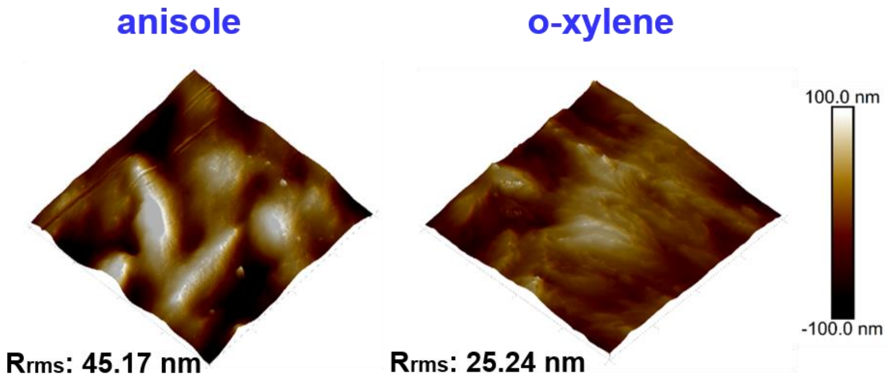

Figure S7. AFM image of $PC_{61}BM$ using anisole and o-Xylene solvent.

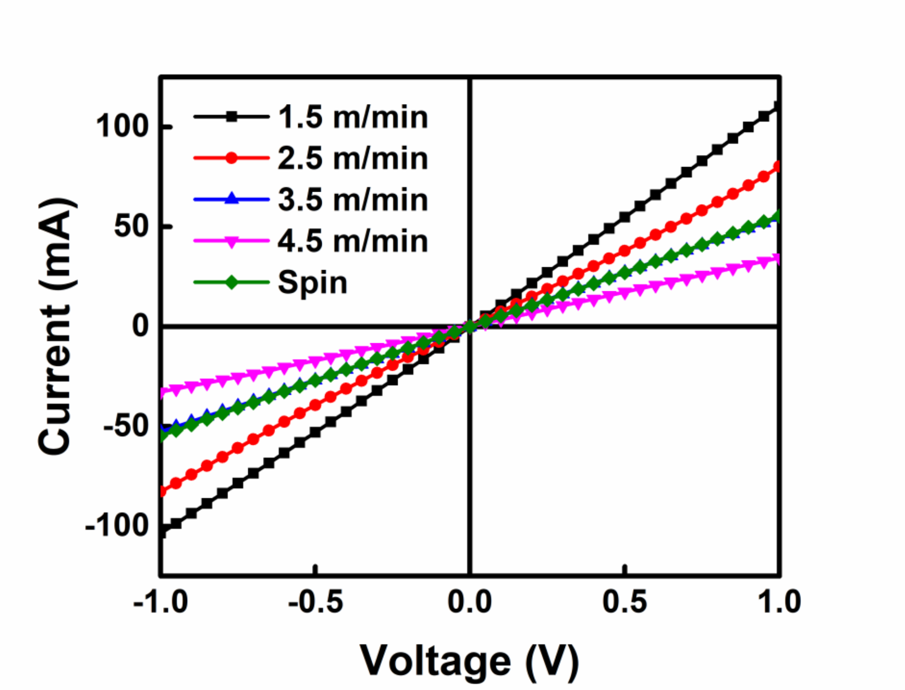

Formatted: Font: Not Bold

Formatted: Font: Not Bold

Formatted: Font: Not Bold

Formatted: Font: Not Bold

Figure S8. The I–V curve of slot-die coated $PC_{61}BM$ film with different moving rate and spin coated $PC_{61}BM$ film obtained by linear sweep voltammetry.

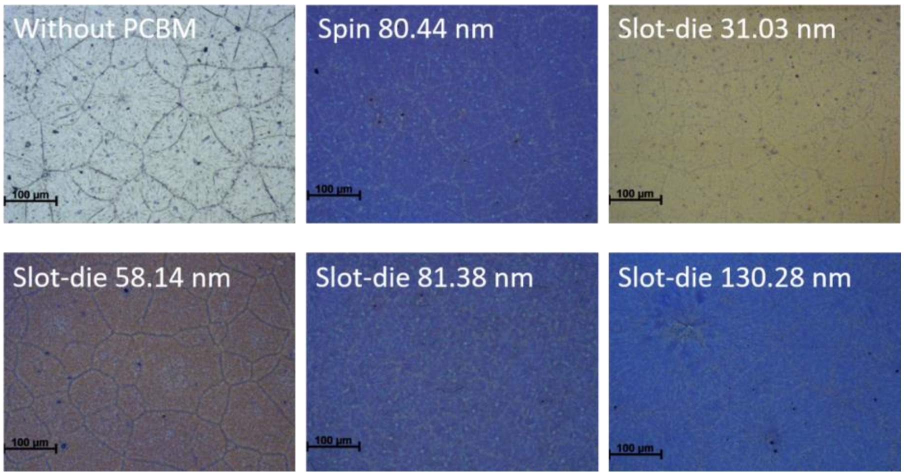

Figure S9. OM images of slot-die coated $PC_{61}BM$ film with different thickness and spin coated $PC_{61}BM$ film.

Table S7. Device performance of slot-die coated PSCs with different WF materials compared with spin coated PSCs.

**Process**	**Work function layer**	V_{OC} **(V)**	J_{SC} **(mA/cm^2)**	**FF (%)**	**PCE (%)**
Slot-die (SD)	PEI	0.97 ±0.04 (1.03)	16.51 ±1.86 (17.40)	51.54 ±5.16 (61.14)	8.38 ±1.75 (11.03)
	TBAOH	1.02 ±0.02 (1.07)	16.47 ±0.84 (18.69)	68.48 ±3.75 (69.17)	11.62 ±0.97 (13.85)
Spin (SP)	PEI	1.03 ±0.02 (1.06)	18.69 ±1.18 (18.89)	67.03 ±5.30 (73.35)	13.03 ±1.73 (15.73)
	TBAOH	1.02 ±0.01 (1.04)	17.41 ±0.96 (18.89)	66.20 ±4.15 (70.46)	11.85 ±1.25 (13.95)

Formatted: Font: Not Bold
Formatted: Font: Not Bold
Formatted: Font: Not Bold
Formatted: Font: Not Bold
Formatted: Font: Not Bold
Formatted: Font: Not Bold
Formatted: Font: Not Bold
Formatted: Font: Not Bold
Formatted: Font: Not Bold
Formatted: Font: Not Bold
Formatted: Font: Not Bold
Formatted: Font: Not Bold
Formatted: Font: Not Bold
Formatted: Font: Not Bold
Formatted: Font: Not Bold
Formatted: Font: Not Bold

Table S8. Device performance of standard perovskite solar cell and 4 cm^2 substrate area module. The J_{SC} and PCE are performed under active area.

Device	V_{OC} (V)	J_{SC} (mA/cm^2)	FF (%)	PCE (%)
Standard cell ($0.09\ cm^2$)	1.03 ±0.02 (1.00)	18.51 ±0.90 (20.34)	62.86 ±4.91 (68.03)	12.03 ±1.08 (13.86)
Module ($0.75\ cm^2$)	1.99 ±0.06 (2.11)	8.92 ±0.87 (10.10)	56.69 ±2.58 (63.30)	10.03 ±1.35 (13.54)

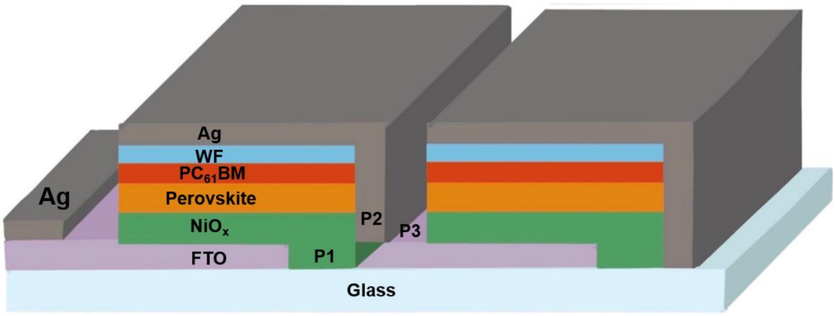

Figure S10. The schematic diagram of the module. The line widths of P1, P2, and P3 were 50 μm, 100 μm, and 100 μm, respectively.

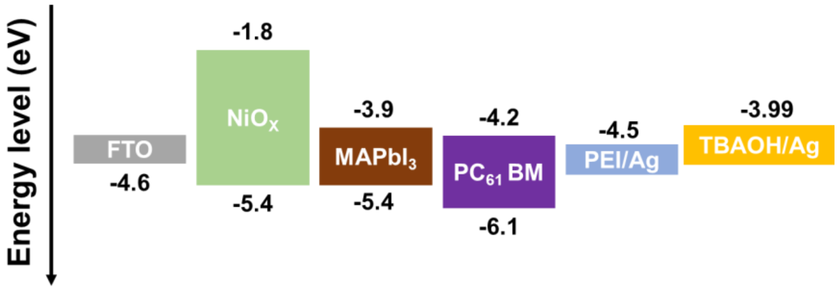

Figure S11. ~~Figure S11.~~ The energy band diagram of the perovskite device.

Formatted: Font: Not Bold

Formatted: Font: Not Bold

Formatted: Font: Not Bold

Formatted: Font: Not Bold

Formatted: Font: Not Bold

Formatted: Font: Not Bold

Formatted: Font: Not Bold

Formatted: Font: Not Bold

Formatted: Centered, Indent: Left: 0 cm, First line: 0 cm, Don't add space between paragraphs of the same style, Line spacing: 1.5 lines
